# Long Noncoding RNAs and Messenger RNAs Expression Profiles Potentially Regulated by ZBTB7A in Nasopharyngeal Carcinoma

**DOI:** 10.1155/2019/7246491

**Published:** 2019-06-11

**Authors:** Fei Liu, Jiazhang Wei, Yanrong Hao, Fengzhu Tang, Wei Jiao, Shenhong Qu, Ning He, Yonglin Cai, Jiao Lan, Yong Yang, Yongli Wang, Min Li, Jingjin Weng, Bing Li, Jinlong Lu, Xing Han

**Affiliations:** ^1^Research Center of Medical Sciences, The People's Hospital of Guangxi Zhuang Autonomous Region, Nanning 530021, China; ^2^Department of Otolaryngology & Head and Neck, The People's Hospital of Guangxi Zhuang Autonomous Region, Nanning 530021, China; ^3^Cancer Center, The People's Hospital of Guangxi Zhuang Autonomous Region, Nanning 530021, China; ^4^Key Laboratory of Nasopharyngeal Carcinoma Etiology and Molecular Mechanism, Wuzhou Red Cross Hospital, Wuzhou 543002, China

## Abstract

Our previous studies showed that ZBTB7A played an important role in promoting nasopharyngeal carcinoma (NPC) progression. However, molecular mechanisms of different levels of ZBTB7A are still unclear. It is necessary to search molecular markers which are closely connected with ZBTB7A. We selected NPC sublines CNE2 with stably transfecting empty plasmid (negative control, NC) and short hair RNA (shRNA) plasmid targeting ZBTB7A as research objectives. Microarray was used to screen differentially expressed long noncoding RNAs (lncRNAs) and messenger RNAs (mRNAs) via shRNA-CNE2 versus NC-CNE2. Quantitative PCR (qPCR) was used to validate lncRNAs and mRNAs from the sublines, chronic rhinitis, and NPC tissues. Bioinformatics was used to analyze regulatory pathways which were connected with ZBTB7A. The 1501 lncRNAs (long noncoding RNAs) and 1275 differentially expressed mRNAs were upregulated or downregulated over 2-fold. Gene Ontology (GO) and Kyoto Encyclopedia of Genes and Genomes (KEGG) pathway analysis revealed that the upregulated or downregulated carbohydrate and lipid metabolisms probably involved in carcinogenicity of shRNA-CNE2 (P-value cut-off was 0.05). In order to find the molecular mechanisms of ZBTB7A, we validated 12 differentially expressed lncRNAs and their nearby mRNAs by qPCR. Most of the differentially expressed mRNAs are closely connected with carbohydrate and lipid metabolisms in multiply cancers. Furthermore, part of them were validated in NPC and rhinitis tissues by qPCR. As a result, NR_047538, ENST00000442852, and fatty acid synthase (FASN) were closely associated with NPC. ZBTB7A had a positive association with NR_047538 and negative associations with ENST00000442852 and FASN. The results probably provide novel candidate biomarkers for NPC progression with different levels of ZBTB7A.

## 1. Introduction

Nasopharyngeal carcinoma (NPC) is an endemic malignant head and neck tumor in southern China [1, 2]. Epstein-Barr virus (EBV) and tumor metastasis related factors are closely connected with NPC progression [3, 4]. The biomarkers of EBV include Epstein-Bar encoded small nuclear RNA (EBER) in tissues, EBV DNA, and anti-EBV antibodies in circulating plasma or serum. All of them are important predictors in early diagnosis and prognosis of NPC. Furthermore, plasma EBV DNA is a better marker in advanced NPC than others [5–7]. However, a few patients have negative EBV DNA in plasma [8, 9]. The negative result indicates a limitation of EBV DNA. In order to improve detection rate of NPC, it is imperative to search novel candidate biomarkers.

ZBTB7A is also named as Pokemon, FBI-1, OCZF, and LRF. It is a critical transcription factor in the poxvirus and zinc finger/broad complex, tramtrack, and bric-a-brac (POZ/BOZ) and Krüppel (POK) family, which can specifically bind DNA through the Krüppel-like C_2_H_2_ zinc fingers and repress transcription by interacting transcriptional cofactors with POZ/BTB domain [10]. Takahiro Maeda [11] firstly discovered that ZBTB7A was a proto-oncogene because it peculiarly repressed the transcription of tumor suppressor alternative reading frame (ARF). Overexpression of ZBTB7A promotes lymphomas progression [11]. It also plays an oncogenic role in non-small cell lung cancer [12], ovarian cancer [13], breast cancer [14], hepatocellular carcinoma [15], and osteosarcoma [16]. However, loss of ZBTB7A shows it is a tumor suppressor in prostate cancer [17], melanoma [18]. The dual roles of ZBTB7A controversially appear in a kind of cancer such as colorectal cancer [19, 20] and gastric cancer [21, 22]. Basing on the complex roles in multiply cancers, ZBTB7A is confirmed to be a significant target of prognosis and therapy [11–22]. It is also an important proto-oncogene in NPC [23–25]. ZBTB7A level of NPC tissues was mostly higher than that of chronic rhinitis tissues [23]. Overexpression of ZBTB7A promoted cell vitality, migration, and invasion (Supplementary Materials, Figure S1 and S2). The tumorigenicity of NPC cell lines CNE2 and CNE3 with transiently decreasing ZBTB7A was weaker than that of cells transfecting empty plasmid [24, 25]. However, progression of NPC cell lines CNE2 and 5-8F was compensatorily promoted when ZBTB7A was stably knocked down [24]. The results indicate that ZBTB7A probably connects with complex pathways including oncogene and tumor suppressor genes.

Long noncoding RNAs (lncRNAs) are non-protein-coding transcripts with more than 200 nucleotides in length [26]. Since lncRNA microarray is continuously developed [27], lncRNAs have been demonstrated to involve in biological processes of cancer [28–30]. Some lncRNAs such as antisense noncoding RNA in the INK4 locus (ANRIL), HOXA transcript at the distal tip (HOTTIP), and differentiation antagonizing non-protein-coding RNA (DANCR) deteriorate NPC development [31–33]. Maternally expressed gene 3 (MEG3) and LINC0086 suppress proliferation of NPC cells [34, 35]. Although the connections between ZBTB7A and lncRNAs are reported in osteosarcoma and non-small cell lung cancer [16, 36, 37], their relationships are still mysterious in NPC.

Therefore, it is meaningful to discover the unknown field. We will screen differentially expressed lncRNAs and messenger RNAs (mRNAs) from NPC cell sublines with stably transfecting empty plasmid and short hair RNA (shRNA) plasmid targeting ZBTB7A by lncRNA microarray. Bioinformatics will be used to search potential connections between ZBTB7A and relevant pathways. Part of them will be selected and validated by qPCR in the sublines, chronic rhinitis, and NPC tissues.

## 2. Methods

### 2.1. Patient Selection

After being approved by patients, 20 chronic rhinitis and 60 NPC tissues were obtained using biopsy (July 2016-February 2017). All of them were preserved in liquid nitrogen. The tissues were confirmed by pathology from the formalin-fixed paraffin wax-embedded samples. The pathological type of NPC was undifferentiated carcinomas of the nasopharyngeal type (UCNT). This study was approved by the ethics committee of The People's Hospital of Guangxi Zhuang Autonomous Region.

### 2.2. Cell Culture

CNE2 and CNE3 were preserved in Research Center of Medical Sciences, The People's Hospital of Guangxi Zhuang Autonomous Region (Nanning, Guangxi, China). CNE2 was established from a patient suffering from NPC in Guangdong Province. The histological type of NPC was a poorly differentiated squamous carcinoma [38]. CNE3 was established from a patient suffering from liver metastatic carcinoma of primary NPC in Guangxi Province. The histological type of liver metastatic carcinoma and CNE3 was a poorly differentiated adenosquamous carcinoma [39], but that of CNE3 turned poorly differentiated adenocarcinoma after 20 years [40]. As a team member of Professor Yi Zeng, Wei Jiao has cultured and detected the cell lines in our hospital [23–25, 41, 42]. 5-8F and 6-10B were kindly provided by Professor Musheng Zeng (State Key Laboratory of Oncology in South China, Sun Yat-Sen University Cancer Center, Guangzhou, Guangdong, China). They were grown in RPMI Medium 1640 basic with 10% fetal bovine serum (Gibco; Thermo Fisher Scientific, Inc., Waltham, MA, USA). NP69 was kindly provided by Professor Sai-Wah Tsao (School of Biomedical Sciences, University of Hong Kong, Hong Kong SAR). NP69 was in Keratinocyte-SFM with 5% Bovine Pituitary Extract and Recombinant Epidermal Growth Factor (Gibco). All of them were in supplementary material. Based on the prior study [24], we used CNE2 sublines with stably transfecting empty plasmid and shRNA plasmid targeting ZBTB7A to screen differentially expressed lncRNAs and mRNAs. The sublines were named as negative control-CNE2 (NC-CNE2) and shRNA-CNE2, of which tumorigenicity was stronger than that of NC-5-8F and NC-6-10B [24]. 

### 2.3. LncRNA Microarray

shRNA-CNE2 and NC-CNE2 were used in lncRNA microarray. Both of them were acquired from three different passages. Total RNA was extracted by TRIZOL (Invitrogen, Carlsbad, CA, USA). RNA quantity and quality were measured by NanoDrop ND-1000 (Thermo Fisher Scientific, Inc.). RNA integrity was assessed by standard denaturing agarose gel electrophoresis. The Arraystar Human lncRNA Microarray V3.0 was designed for the global profiling of human lncRNAs and protein-coding mRNAs. 30,586 lncRNAs and 26,109 mRNAs could be detected by third-generation lncRNA microarray.

### 2.4. RNA Labeling and Array Hybridization

Sample labeling and array hybridization were performed according to the Agilent One-Color Microarray-Based Gene Expression Analysis protocol (Agilent Technology, Santa Clara, CA 95051, USA) with minor modifications. Briefly, mRNA was purified from total RNA after removal of ribosome RNA (rRNA; mRNA-ONLY™ Eukaryotic mRNA Isolation Kit; Epicentre, Madison, WI 53719, USA). Each sample was amplified and transcribed into fluorescent complementary RNA (cRNA) along the entire length of the mRNAs without 3′ bias utilizing a random priming method (Arraystar Flash RNA Labeling Kit; Arraystar, Inc., Rockville, MD 20850, USA). The labeled cRNAs were purified by RNeasy Mini Kit (Qiagen, Düsseldorf, Nordrhein-Westfalen, Germany). The concentration and specific activity of the labeled cRNAs (pmol Cy3/*μ*g cRNA) were measured by NanoDrop ND-1000. 1 *μ*g of each labeled cRNA was fragmented by adding 5 *μ*l 10 x Blocking Agent and 1 *μ*l of 25 x Fragmentation Buffer, then the mixture was heated at 60°C for 30 min, finally 25 *μ*l 2 x GE hybridization buffer was added to dilute the labeled cRNA. 50 *μ*l of hybridization solution was dispensed into the gasket slide and assembled to the lncRNA expression microarray slide. The slides were incubated for 17 h at 65°C in an Agilent Hybridization Oven. The hybridized arrays were washed, fixed, and scanned with using the Agilent DNA Microarray Scanner (part number G2505C; Agilent Technology).

### 2.5. Data Analysis

The Agilent Feature Extraction software (version 11.0.1.1) was used to analyze acquired array images. Quantile normalization and subsequent data processing were performed with using the GeneSpring GX v12.1 software package (Agilent Technologies). Differentially expressed lncRNAs and mRNAs between the two groups were identified using fold-change >2 as the cut-off. Hierarchical clustering and combined analysis were performed using homemade scripts. Gene ontology (GO) and pathway analysis were performed in the standard enrichment computation method.

### 2.6. Validation of the Differentially Expressed LncRNAs and MRNAs by QPCR

The total RNA of NC-CNE2 and shRNA-CNE2 cells, chronic rhinitis, and NPC tissues was extracted by TRIZOL (Invitrogen) and reversely transcribed by SuperScript ™ III Reverse Transcriptase Kit (Invitrogen). 12 lncRNAs and their nearby mRNAs were selected basing on data analysis. All of them were differentially expressed. The quantitative PCR (qPCR) was executed with 2 × PCR master mix (Arraystar) by ViiA 7 qPCR System (Applied Biosystems; Thermo Fisher Scientific, Inc.). Specific primers of the lncRNAs and nearby mRNAs were designed by Primer 5.0 (Table 1). The reaction program consisted of an initial denaturation step at 95°C for 10 minutes; denaturation at 95°C for 10 seconds, and annealing at 60°C for 60 seconds for 40 cycles; the dissociation stage at 95°C for 10 seconds, 60°C for 1 minute, 95°C for 15 seconds, and 60°C for 15 seconds. Glyceraldehyde-3-phosphate dehydrogenase (GAPDH) was used as an internal control. The experiments were performed in triplicate. The fold-change of the lncRNAs and mRNAs were calculated by the 2 Delta-delta cycle threshold (Ct) method.

### 2.7. Statistical Analysis

Statistical analysis was performed by SPSS 13.0 and sigmaPlot 12.5 (SPSS Inc., Chicago, IL, USA), GraphPad Prism 5.0 (GraphPad Software, San Diego, CA, USA). The results of the assays were presented as mean ± Standard Deviation (SD). Statistical differences between two groups were evaluated with independent samples t-test. In GO analysis, Fisher's exact test was used to search more overlap between the differentially expressed list and the GO annotation list than that of being expected by chance. In scatter plot of tissues by qPCR, Mann Whitney U test was used if the variances were significantly different through F test. P<0.05 was considered to be statistically significant.

## 3. Results

### 3.1. Differentially Expressed LncRNAs and MRNAs in NPC Cells with Low Expression of ZBTB7A

**Figure 1:**
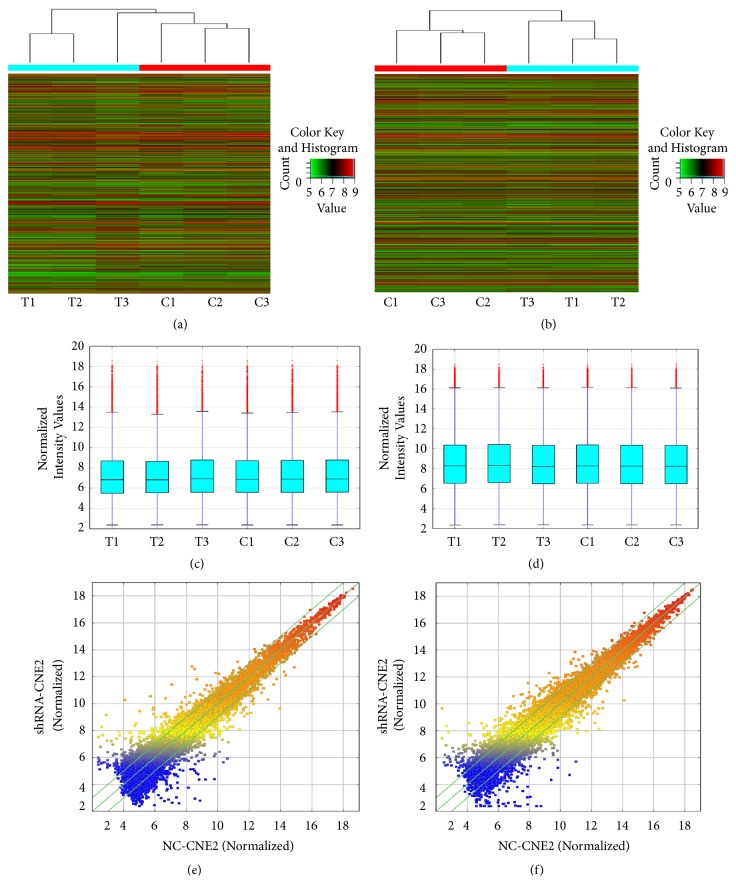
*RNA expression profiles in shRNA-CNE2 and NC-CNE2 cells.* Hierarchical clustering indicated (a) lncRNA and (b) mRNA profiles. Box plot of (c) lncRNA or (d) mRNA profiles was a traditional method for visualizing separately the distribution of dataset. Scatter plot of (e) lncRNA or (f) mRNA profiles was a convenient visualization for assessing the variation of chips. T1-T3 meant test groups which were stably transfected with shRNA plasmid targeting ZBTB7A. C1-C3 meant negative control groups which were stably transfected with blank plasmid. ‘Red' showed relatively high expression. ‘Green' showed relatively low expression. The values of the x- and y-axes were the average normalized signal values of NC-CNE2 and shRNA-CNE2 in the scatter plot (log⁡2 scaled).

**Figure 2:**
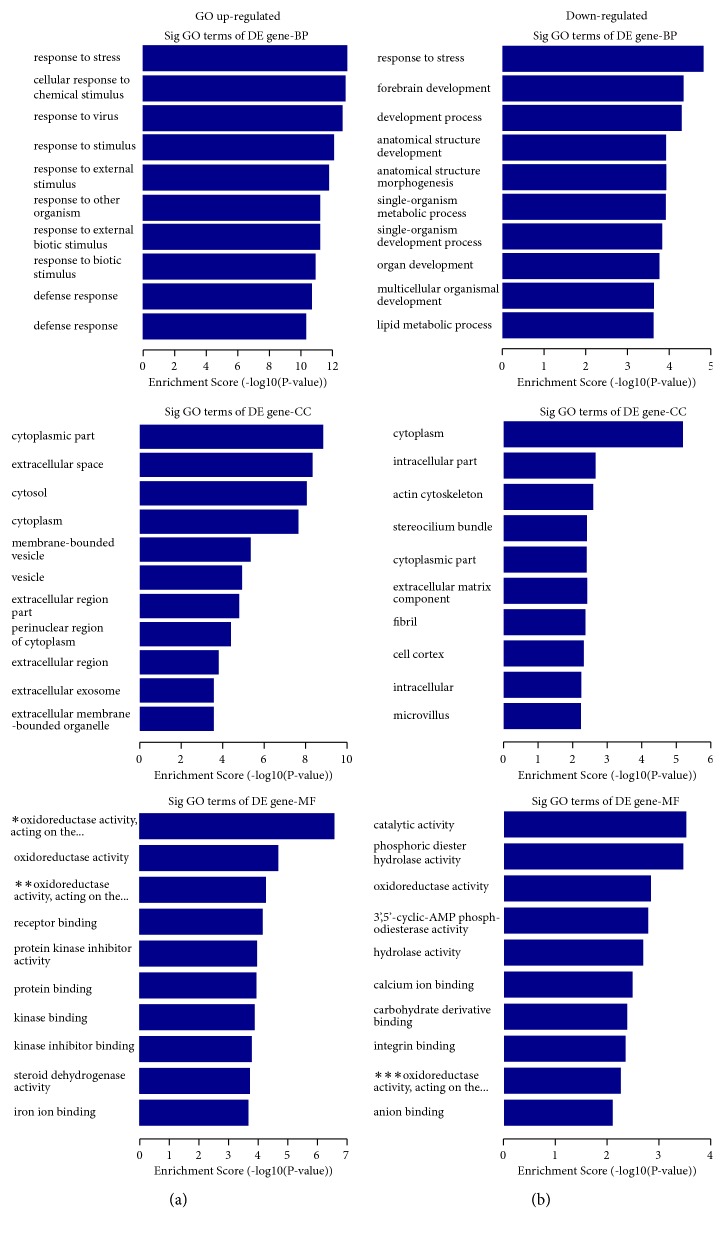
*GO (gene ontology) analysis.* (a) GO upregulated. (b) GO downregulated. The P value denoted the significance of GO terms enrichment in the DE mRNAs. The lower the p-value, the more significant the GO Term (P value ≤0.05 was recommended). DE, differentially expressed; BP, biological process; CC, cellular component. MF, molecular function. *∗* It meant oxidoreductase activity, acting on the CH-CH group of donors, NAD or NADP as acceptor. *∗∗* It meant oxidoreductase activity, acting on the CH-CH group of donors. *∗∗∗* It meant oxidoreductase activity, acting on the aldehyde or oxo group of donors.

After quantile normalization of the raw data, the expression profiles of 23947 lncRNAs and 21058 mRNAs were obtained from shRNA-CNE2 and NC-CNE2 (Figure 1). Cluster analysis showed some lncRNAs and mRNAs were differentially expressed via shRNA-CNE2 versus NC-CNE2 (Figures 1(a) and 1(b)). Box plot showed the distribution of normalized intensity values of test and control groups was generally symmetrical (Figures 2(c) and 2(d)). Scatter plot figuratively showed relative folds of 1501 lncRNAs (738 upregulated and 763 downregulated) and 1276 mRNAs (679 upregulated and 597 downregulated), which were over 2-fold. The number of differentially expressed lncRNAs and mRNAs was listed. We divided them into 3 groups according to different fold-change. There were 2-5-fold-change, 5-10-fold-change, and more than 10-fold-change. 77 lncRNAs and 44 mRNAs were over 10-fold-change (Table 2).

### 3.2. GO Analysis

GO is a system of standard classification of gene function, including biological process (BP), cellular component (CC), and molecular function (MF). The results showed the significant GO terms of differentially expressed genes. We found that the highest enriched GO terms of upregulated mRNAs were response to stress (GO: BP), cytoplasmic part (GO: CC), and oxidoreductase activity (GO: MF) (Figure 2(a)). The highest enriched GO terms of downregulated mRNAs were response to stress (GO: BP), cytoplasm (GO: CC), and catalytic activity (GO: BF) (Figure 2(b)). The results indicated some differentially expressed lncRNAs and relevant mRNAs probably involved in the activities.

### 3.3. KEGG Pathway Analysis

**Figure 3:**
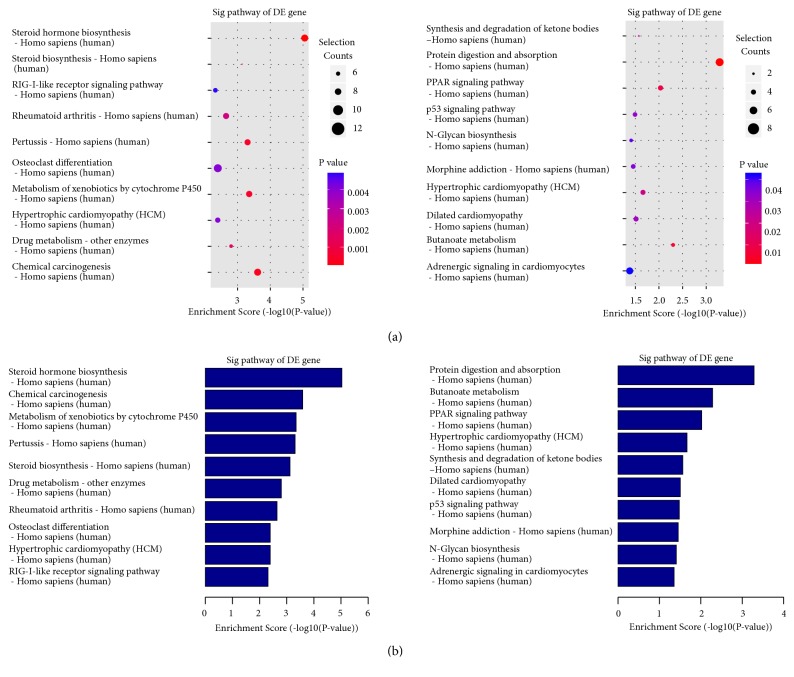
*KEGG pathways in shRNA-CNE2 compared to NC-CNE2.* (a) The most significantly enriched KEGG (Kyoto Encyclopedia of Genes and Genomes) pathways were illustrated by bubbles. The y-axis indicated the enrichment factors, which refers to the upregulated (left) and downregulated (right) differentially expressed mRNA number to the total mRNAs in a certain pathway. The size of bubble indicated mean number of mRNA enriched in a given pathway. The colour of bubble indicated P value (adjusted* p* value). (b) Bar plots represented enriched KEGG pathways associated with upregulated (left) and downregulated (right) DE mRNAs. The y-axis indicated average change fold of the implicated mRNAs. Sig, significant; DE, differentially expressed.

Kyoto Encyclopedia of Genes and Genomes (KEGG) is a database resource for knowing high-level biological functions and utilities. KEGG pathways were constructed to better know the biological pathways and search the molecular mechanisms of NPC progression. The results showed significant pathways of differential genes. KEGG pathways connected with upregulated (Figures 3(a) and 3(b), left) mRNAs and downregulated (Figures 3(a) and 3(b), right) differentially distributed mRNAs. The top upregulated pathway was steroid hormone biosynthesis. Steroid is a kind of lipid. The top downregulated pathway was protein digestion and absorption. The third downregulated pathway was peroxisome proliferators-activated receptor (PPAR) signaling pathway, which included important lipid metabolism. 463 differentially expressed mRNAs were closely associated with carbohydrate and lipid metabolisms. The pathways of lipid metabolism probably were more meaningful than those of carbohydrate metabolism because of more enrichment scores in lipid pathways. In upregulated pathways, the scores of steroid hormone biosynthesis and steroid biosynthesis were 5.04301 and 3.129842. In downregulated pathways, the score of PPAR signaling pathway was 2.035416 (all p<0.05) (Table 3).

### 3.4. Signal Pathway Analysis

**Figure 4:**
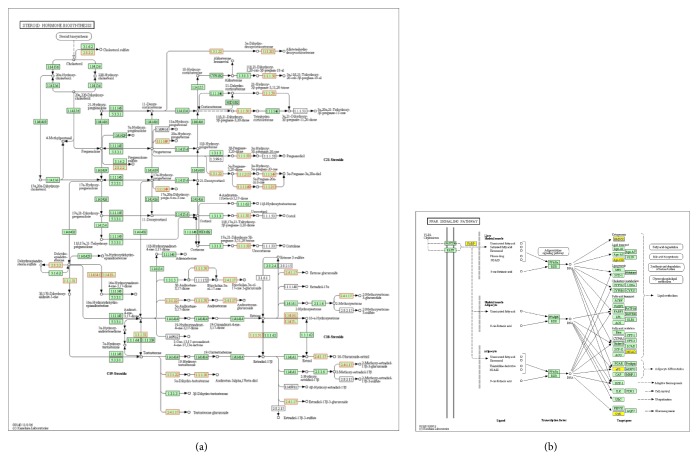
*The differentially expressed mRNAs in important ranked pathways.* (a) Upregulated mRNAs in steroid hormone biosynthesis. 2.8.2.2, SULT2B1 (sulfotransferase family 2B member 1); 1.3.1.22, SRD5A3 (steroid 5 alpha-reductase 3); 1.1.1.213, AKR1C2 (aldo-keto reductase family 1 member C2); 1.1.1.50, AKR1C4; 1.1.1.149, AKR1C1; 1.1.1.51, AKR1C3; 1.14.14.1, CYP3A5 (cytochrome P450 family 3 subfamily A member 5); 1.14.13.-, CYP3A4 (cytochrome P450 family 3 subfamily A member 4); 2.4.1.17, UGT1A6, (UDP glucuronosyltransferase family 1 member A6). (b) Downregulated mRNAs in PPAR signaling pathway. PPAR, peroxisome proliferators-activated receptor; FABP, fatty acid binding protein; aP2, FABP4; HMGCS2, 3-hydroxy-3-methylglutaryl-CoA synthase 2; Apo-CIII, apolipoprotein C3; MCAD, ACADM (acyl-CoA dehydrogenase medium chain); GyK, GK (glycerol kinase). The regulated mRNAs in shRNA-CNE2 compared to NC-CNE2 were marked in coloured boxes. Red box indicated an upregulated mRNA; yellow box indicated a downregulated mRNA; green box indicated that no significant difference was observed.

Steroid hormone biosynthesis was markedly activated in NPC cells with low expression of ZBTB7A. Aldo-keto reductase family 1 member C3 (AKR1C3) was markedly upregulated in the pathway. PPAR signaling pathway was markedly suppressed in the cells. Fatty acid binding protein 4 (FABP4) was markedly downregulated. The differentially expressed mRNAs of the pathways were associated with lipid metabolism, such as AKR1C3 and FABP4 (Figure 4).

### 3.5. QPCR Validation

**Figure 5:**
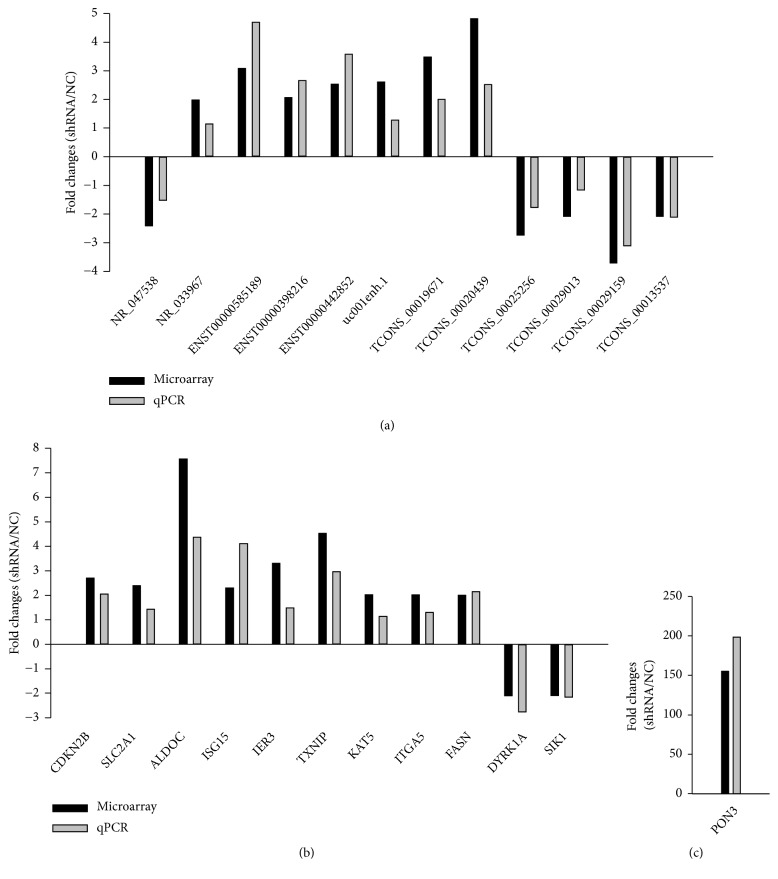
*Validation of lncRNA microarray by qPCR through shRNA-CNE2 versus NC-CNE2.* (a) 12 differentially expressed lncRNAs and (b), (c) nearby mRNAs were validated by qPCR through shRNA-CNE2 versus NC-CNE2. The heights of the columns in the charts represent the mean expression values of fold-change. CDKN2B, cyclin-dependent kinase inhibitor 2B; SLC2A1, solute carrier family 2 member 1, also named glucose transporter 1 (GLUT1); ALDOC (aldolase C); ISG15 (interferon-stimulated gene 15); IER3 (immediate early response 3); TXNIP (thioredoxin-interacting protein); KAT5 (lysine acetyltransferase 5); ITGA5 (integrin subunit alpha 5); FASN (fatty acid synthase); DYRK1A (dual specificity tyrosine phosphorylation regulated kinase 1A); SIK1 (salt inducible kinase 1); PON3 (paraoxonase 3).

**Figure 6:**
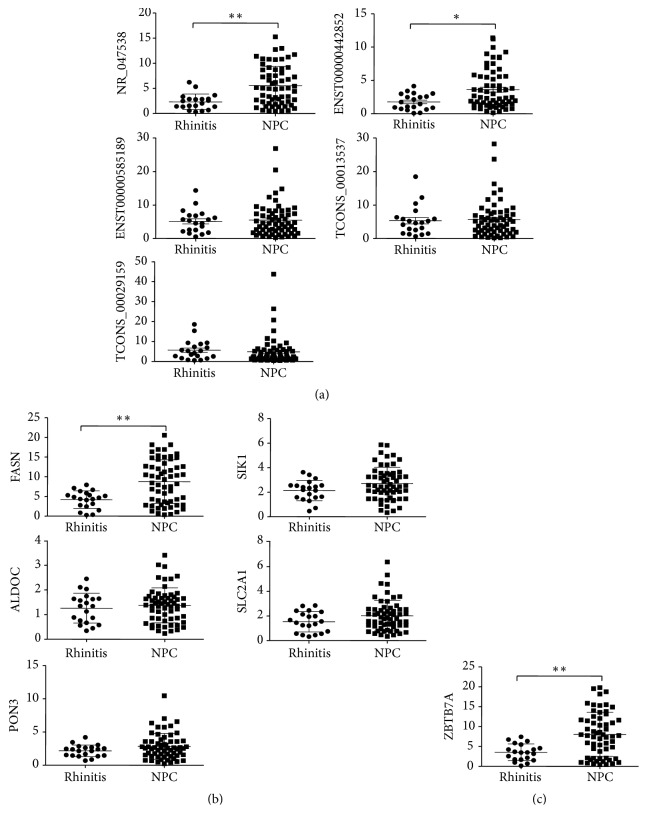
*Validation of microarray data by qPCR in NPC and chronic rhinitis tissues.* The expressions of (a) 5 differentially expressed lncRNAs, (b) 5 differentially expressed mRNAs, and (c) ZBTB7A were detected by qPCR in NPC and rhinitis tissues. *∗* p<0.05, *∗∗* p<0.01.

**Figure 7:**
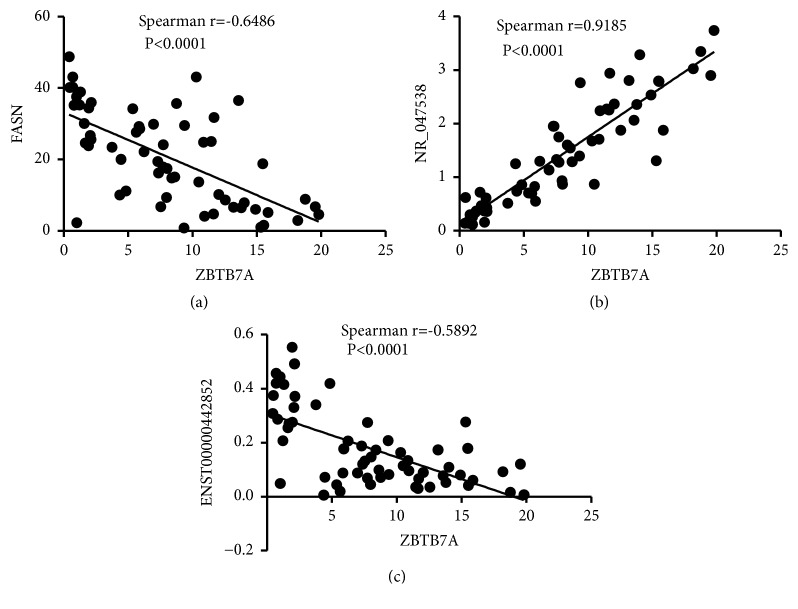
*ZBTB7A were separately connected with NR_047538, FASN, and ENST00000442852 by scatter plot.* ZBTB7A had a positive association with (a) FASN and negative associations with (b) NR_047538 and (c) ENST00000442852. p<0.05 was considered to be statistically significant.

lncRNAs mainly include antisense lncRNAs, enhancer lncRNAs, and long intergenic noncoding RNAs (lincRNAs). They have different biological functions through regulating nearby mRNAs [43–45]. In order to validate authenticity and reliability of lncRNA microarray, 12 differentially expressed lncRNAs and their nearby mRNAs were detected by qPCR. The differentially expressed mRNAs are closely connected with carbohydrate and/or lipid metabolisms of cancers, which means the lncRNAs probably interact with cancers via the mRNAs. The results of qPCR were consistent with those of lncRNA microarray through shRNA-CNE2 versus NC-CNE2. All of them have shown the same upregulation or downregulation trend (Figure 5). 6 lncRNAs (NR_033967, ENST00000398216, uc001enh.1, TCONS_00019671, TCONS_00025256, TCONS_00029013) and 1 mRNA (integrin subunit alpha 5, ITGA5) would not be used in next assay because the results of qPCR were not considered to be statistically significant (P>0.05). The other differentially expressed lncRNAs (NR_047538, ENST00000585189, ENST00000442852, TCONS_00020439, TCONS_00029159, TCONS_00013537) and mRNAs (cyclin-dependent kinase inhibitor 2B (CDKN2B), solute carrier family 2 member 1 (SLC2A1), aldolase C (ALDOC), interferon-stimulated gene 15 (ISG15), immediate early response 3 (IER3), thioredoxin-interacting protein (TXNIP), lysine acetyltransferase 5 (KAT5), FASN (fatty acid synthase), dual specificity tyrosine phosphorylation regulated kinase 1A (DYRK1A), salt inducible kinase 1 (SIK1), and paraoxonase 3 (PON3)) were continuingly validated by qPCR in chronic rhinitis and NPC tissues (Table 4). The results showed that NR_047538, ENST00000442852, fatty acid synthase (FASN), and ZBTB7A were closely connected with NPC (Figure 6). ZBTB7A had a positive association with NR_047538 and negative associations with ENST00000442852 and FASN (Figure 7).

## 4. Discussion

lncRNA microarrays have been used in studies of multiple cancers [27, 46, 47]. Differentially expressed lncRNAs and mRNAs can be found by the method, which reveals some lncRNAs are important regulatory factors in melanoma, renal tumor, hepatocellular carcinoma, and colorectal cancer [48–51]. lncRNA ANRIL, also called NR_047538, promotes NPC progression via reprogramming cell glucose metabolism [31]. lncRNA HOTAIR promotes tumorigenesis of NPC via upregulating FASN, which is a pivotal enzyme of lipid metabolism [52]. Therefore, the technique is effectively applied in NPC study of exploring possible connections between ZBTB7A and differentially expressed lncRNAs.

Comparing to protein levels of ZBTB7A of NPC cell lines CNE2, CNE3, 5-8F, and 6-10B (Supplementary Materials, Figure S1(a)), we selected CNE2 and 5-8F with high expression of ZBTB7A as the objectives of stably knocking down ZBTB7A [24]. Then we selected CNE3 and 6-10B with low expression of ZBTB7A as the objectives of transfecting with ZBTB7A plasmid. The cells were transfected with plasmids and selected by G418. The best concentrations were 200 *μ*g/ml (6-10B) and 230 *μ*g/ml (CNE3). The concentration was, respectively, reduced to 100 *μ*g/ml and 115 *μ*g/ml after 10 days. The clone cells were stably cultured during 15 passages. The cells stably transfected with plasmids were named NC-6-10B, ZBTB7A-6-10B, NC-CNE3, and ZBTB7A-CNE3. The protein levels of ZBTB7A-6-10B and ZBTB7A-CNE3 were higher than those of control groups (Supplementary Materials, Figure S1 (b)). ZBTB7A-6-10B and ZBTB7A-CNE3 separately had stronger tumorigenicity than NC-6-10B and NC-CNE3 by 3-(4,5-dimethyl-2-thiazolyl)-2,5-diphenyl-2-H-tetrazolium bromide (MTT) and transwell assays (Supplementary Materials, Figure S2). The results of transiently and stably knocking down ZBTB7A in NPC cell line CNE2 were opposite [24]. We hypothesize that some important pathways vicariously maintain carcinogenicity of NPC cells. Therefore, it is a good method to search unknown pathways of connecting with ZBTB7A by lncRNA microarray.

CNE2 cells have specific characteristic of NPC because they are susceptible to EBV infection in vitro, while Hela cells are resistant [53]. They also have specific biomarkers and pathways of NPC [27, 54, 55]. Considering the differentially stable carcinogenicity, NC-CNE2 and shRNA-CNE2 were suitable models for screening differentially expressed lncRNAs and mRNAs NPC by lncRNA microarray.

As a result, some differentially expressed mRNAs indicated that ZBTB7A may connect with oncogenic and oncosuppressive pathways (Supplementary Materials, Figure S3 and Table S1). Loss of ZBTB7A caused upregulation of some oncogenes such as matrix metalloproteinase-10 (MMP10) [56] and lysyl oxidase-like 2 (LOXL2) [57], and downregulation of some tumor suppressor genes such as suppressor of cytokine signalling (SOCS3) [58] and fibulin 1 (FBLN1) [59]. The results indicate that ZBTB7A possibly plays an oncosuppressive role. However, loss of ZBTB7A also caused downregulation of some oncogenes such as myosin light chain kinase (MYLK) [60] and chloride intracellular channel 5 (CLIC5) [61] and upregulation of some tumor suppressor genes such as interleukin-24 (IL24) [62] and arachidonate 15-lipoxygenase, type B (ALOX15B) [63]. The results indicate that ZBTB7A possibly plays an oncogenic role. Interestingly, we find that some genes have opposite effects in carcinogenesis, such as cellular retinoic acid binding protein 2 (CRABP2) [64, 65], epithelial membrane protein 3 (EMP3) [66, 67], transglutaminase 2 (TGM2) [68, 69], Yes associated protein 1 (YAP1) [70, 71], CDKN2A interacting protein (CDKN2AIP) [72], and BRCA2 and CDKN1A interacting protein (BCCIP) [73]. The genes can promote or inhibit cells growth in different conditions or stages of cancer (Supplementary Materials, Figure S3 and Table S1).

We went on disclosing that carbohydrate and lipid metabolisms probably involved in NPC progression through KEGG analysis. In order to search the molecular mechanisms of ZBTB7A, we selected 12 differentially expressed lncRNAs and their nearby mRNAs to validate. 12 differentially expressed mRNAs included FASN, CDKN2B, SLC2A1, ALDOC, ISG15, IER3, TXNIP, KAT5, ITGA5, DYRK1A, SIK1, and PON3. Basing on the bioinformatics analysis, the lncRNAs and mRNAs are possibly associated with cancer cells progression via carbohydrate or lipid pathway. In order to exclude the contaminated possibility by Hela cell, parts of them were validated in NPC and rhinitis tissues by qPCR. lncRNA NR_047538, lncRNA ENST00000442852, and fatty acid synthase (FASN) were closely associated with NPC. ZBTB7A had a positive association with NR_047538, while having negative associations with ENST00000442852 and FASN. The results provide novel candidate biomarkers for NPC progression with different levels of ZBTB7A.

LncRNA NR_047538 and FSAN were important markers and regulatory factor in NPC [31, 74]. LncRNA NR_047538 can promote NPC progression by upregulating GLUT1 expression [31]. It is also associated with lipid pathways [75]. Sex determining region Y-box 2 gene (SOX2) induces proliferation of NPC cells through activating lncRNA NR_047538 [76]. Downregulation of lncRNA NR_047538 inhibits NPC tumorigenicity and enhances the efforts of radiotherapy and chemotherapy via regulating microRNA 125a and let-7a [77, 78]. FASN is an important enzyme of lipogenesis. It promotes NPC progression through remarkably providing endogenous fatty acid [52]. Interestingly, we found that sterol regulatory element binding transcription factor 1 (SREBF1) also was upregulated by lncRNA microarray and qPCR (Supplementary Materials, Figure S4 and Table S1). SREBF1 is the transcript of sterol regulatory element binding protein 1 (SREBP1). Activated SREBP1 enters nucleus and transcribes the genes of lipid metabolism such as FASN [79]. EBV-encoded latent membrane protein 1 (LMP1) induces cell proliferation and NPC metastasis via activating SREBP1 and its downstream FASN [80].

The potential biomarker lncRNA ENST00000442852 has not been reported. We speculate it may be connected with the nearby mRNA IER3, which is also called immediate early response gene X-1 (IEX-1). It is immediately regulated by transcriptional factors, inflammatory cytokines, growth factors, and so on [81]. It is connected with poor or good prognosis in different types of cancers [82, 83]. Therefore, the functions and characteristics of lncRNA ENST00000442852 will be explored in NPC.

Although SLC2A1, CDKN2B, and ISG15 are closely connected with NPC progression in other studies [31, 84, 85], our results do not show the positive results. The quantity of 20 chronic rhinitis and 60 NPC tissues is limited. The genotyping of Guangxi population probably is different from that of other region. The protein expression of the genes should be validated by Western blot or immunohistochemistry in future because of the difference between mRNA and protein expression of the same gene.

## 5. Conclusions

Above all, the results showed the changes of the differentially expressed lncRNAs and mRNAs with stable loss of ZBTB7A expression in shRNA-CNE2 cells. The results may provide potential biomarkers for NPC progression with different levels of ZBTB7A, such as lncRNA NR_047538, lncRNA ENST00000442852, and FASN. In the future, we will deeply dig the connections between the lncRNAs/mRNAs and ZBTB7A in NPC.

## Figures and Tables

**Table 1:** The list of specific primers of 12 differentially expressed lncRNAs and nearby mRNA designed utilizing primer 5.0.

Number	Seqname	Prime Sequence
	(GeneSymbol)	
1*∗*	NR_047538	F:5′CCCTTATTTTATTCCTGGCTCC3′
	(CDKN2B-AS1)	R:5′CGGATAGAGCAATGAGATGACC3′
	*NM_004936*	*F:5*′*AGCGAAACACAGAGAAGCGG3*′
	*(CDKN2B)*	*R:5*′*CAGCAGACATTGGAGTGAACG3*′
2*∗*	NR_033967	F:5′ACAGCAGAAAATGCCCACG3′
	(SLC2A1-AS1)	R:5′GGTTCCCAAATTGTTCCTACC3′
	*NM_006516*	*F:5*′*ATCATCGGTGTGTACTGCGG3*′
	*(SLC2A1)*	*R:5*′*GTTCTCCTCGTTGCGGTTG3*′
3*∗*	ENST00000585189	F:AGCTCTGGCATCTCCAGTCA
	(RP11-192H23.5)	R:CAGATCCTGCCGTTGTTACC
	*NM_005165*	*F:5*′*CAGGATAAGGGCATCGTCG3*′
	*(ALDOC)*	*R:5*′*GCTGGCAGATACTGGCATAAC3*′
4*∗*	ENST00000398216	F:5′ATGGGTTGAGGGTCTGTTGT3′
	(RP11-54O7.2)	R:5′TTTTCTGAGTGGCCTGGGT3′
	*NM_005101*	*F:5*′*CAGCTCCATGTCGGTGTCAG3*′
	*(ISG15)*	*R:5*′*GAGGTTCGTCGCATTTGTCC3*′
5*∗*	ENST00000442852	F:5′TGTCTTCATTAGTCTGGTCCTCC3′
	(XXbac-BPG27H4.8)	R:5′CCTTTCTCCTGGTCATTTGTTC3′
	*NM_003897*	*F:5*′*CACTCCCCAAAAAGAATCCG3*′
	*(IER3)*	*R:5*′*CTCCGCTGTAGTGTTCTGAGTTC3*′
6*∗*	uc001enh.1	F:5′CGAGCAATGTTCTGTAGTTGTC3′
	(AX747132)	R:5′GTAAGAATAATATGCCTGGGAA3′
	*NM_006472*	*F:5*′*CTGATGGGCGGGTGTCTGT3*′
	*(TXNIP)*	*R:5*′*GGCAAGGTAAGTGTGGCGG3*′
7*∗*	TCONS_00019671	F:5′CAGAGGAAAATAGATGCGACAG3′
	(XLOC_009475)	R:5′TGCGTTCTTAGCCGTGATG3′
	*NM_182709*	*F:5*′*CTCTACCTGTGCGAGTTCTGC3*′
	*(KAT5)*	*R:5*′*TGTGGAAGCCCTTACAGTCATAC3*′
8*∗*	TCONS_00020439	F:5′TTCCACCAAAAGCCAGCAC3′
	(XLOC_009769)	R:5′GACTGGGGTTGACCACTCTGT3′
	*NM_002205*	*F:5*′*GTGACTACTTTGCCGTGAACCA3*′
	*(ITGA5)*	*R:5*′*GAGATGAGGGACTGTAAACCGA3*′
9*∗*	TCONS_00025256	F:5′CTTGGGAATAGGGTCATCG3′
	(XLOC_012590)	R:5′GCTTCTCCTGTGTGTCTGTCTC3′
	*NM_004104*	*F:5*′*TCCGAGTCTCCTGACCACTACCT3*′
	*(FASN)*	*R:5*′*GCAGCACCACATCCTCAAACA3*′
10*∗*	TCONS_00029013	F:5′TCAAAGGAGCAAGGGGAACT3′
	(XLOC_013936)	R:5′ CGCACTTAGCAACCATCACA3′
	*NM_101395*	*F:5*′*CTGAGCAGACAGGCTGGTATT3*′
	*(DYRK1A)*	*R:5*′*ACAGGTTATCGGCAGAGGTAG3*′
11*∗*	TCONS_00029159	F:5′GCAAAATGGGGAGGATGAGGT3′
	(XLOC_014103)	R:5′ACCGTCTTGAGGCAGGTGTT3′
	*NM_173354*	*F:5*′*CTGAGCAGACAGGCTGGTATT3*′
	*(SIK1)*	*R:5*′*ACAGGTTATCGGCAGAGGTAG3*′
12*∗*	TCONS_00013537	F:5′ GATGGGGATAGGAGGTTGGA3′
	(XLOC_006176)	R:5′GGTGAATTGGGAGATGGAGAA3′
	*NM_000940*	*F:5*′*TTTTACCAACTCCCTCCTGTCA3*′
	*(PON3)*	*R:5*′* TGCCCAACTGTATCACCTTCA3*′
13*∗∗*	NM_002046.5	F:5′GGGAAACTGTGGCGTGAT3′
	(GAPDH)	R:5′GAGTGGGTGTCGCTGTTGA3′

Seqname: the sequence identifier; GeneSymbol: the symbol of the lncRNA or mRNA.

*∗* In Number 1-Number 12, 12 differentially expressed lncRNAs and their nearby mRNAs were validated by qPCR. Italic scripts represented sequence name, gene symbol, and prime sequences of the mRNAs.

*∗∗* GAPDH was used as an internal reference control.

**Table 2:** The number of differentially expressed lncRNAs and mRNAs (shRNA-CNE2 *vs*. NC-CNE2, p<0.05).

Regulation	Fold-change			Total
	2-5	5-10	>10	
lncRNA				
Up-regulation	666	38	34	738
Down-regulation	662	58	43	763
mRNA				
Up-regulation	598	67	14	679
Down-regulation	507	60	30	597

**Table 3:** Top up-regulated and down-regulated KEGG pathways of carbohydrate and lipid metabolisms (shRNA-CNE2 *vs*. NC-CNE2, p<0.05).

Pathway ID	Definition	Count	Enrichment	Fisher-P
			Score	Value
Up-regulated				
hsa00140	Steroid hormone biosynthesis	58	5.04301	0.00000906
hsa00100	Steroid biosynthesis	46	3.129842	0.00074158
hsa05205	Proteoglycans in cancer	204	1.970321	0.01070727
hsa00053	Ascorbate and aldarate metabolism	27	1.744802	0.01799693
Down-regulated				
hsa03320	PPAR signaling pathway	69	2.035416	0.00921689
hsa00072	Synthesis and degradation of	10	1.570479	0.02688568
	ketone bodies			
hsa00510	N-Glycan biosynthesis	49	1.40886	0.03900677

**Table 4:** The p values of the differentially expressed lncRNAs and nearby mRNAs by microarray and qPCR.

lncRNA	Types*∗*	Microarray	qPCR	mRNAs	Microarray	qPCR
	P value	PCC				
NR_047538	Antisense	0.01588	0.02259	CDKN2B	0.00328	0.01983
NR_033967	Antisense	0.00769	0.4833*∗∗*	SLC2A1	0.01188	0.00221
ENST00000585189	Antisense	0.001	0.00173	ALDOC	0.00156	0.00313
ENST00000398216	Enhancer	0.00246	0.065*∗∗*	ISG15	0.00059	0.00002
ENST00000442852	Enhancer	0.00008	0.00145	IER3	0.00065	0.00324
uc001enh.1	Enhancer	0.01706	0.06825*∗∗*	TXNIP	0.00623	0.0168
TCONS_00019671	lincRNA	0.00055	0.10619*∗∗*	KAT5	0.00278	0.03497
TCONS_00020439	lincRNA	0.000001	0.00359	ITGA5	0.00372	0.0562*∗∗*
TCONS_00025256	lincRNA	0.00052	0.23031*∗∗*	FASN	0.00031	0.00072
TCONS_00029013	lincRNA	0.02408	0.77654*∗∗*	DYRK1A	0.03928	0.0035
TCONS_00029159	lincRNA	0.00378	0.01099	SIK1	0.00857	0.00126
TCONS_00013537	lincRNA	0.04786	0.02526	PON3	0.0000002	0.00021

*∗* According to the relationships of lncRNAs and nearby mRNAs, the types of lncRNAs were generally divided into antisense lncRNA, enhancer lncRNA, lincRNA (long intergenic noncoding RNA).

*∗∗* p value >0.05

## Data Availability

The data used to support the findings of this study are included in the link of https://drive.google.com/drive/folders/12uRcwskYElRlXr4j_rdVRpO4n61yNFLR?usp=sharing.

## Supplementary Materials

Supplementary 1 Figure S1. The protein levels of ZBTB7A in NPC cell lines.

Supplementary 2 Figure S2. The vitality, migration, and invasion of 6-10B and CNE3 stably transfected cells with ZBTB7A overexpression.

Supplementary 3 Figure S3. Screening and validation of 14 differentially expressed mRNAs by microarray and qPCR.

Supplementary 4 Figure S4. Screening and validation of differentially expressed SREBF1 by microarray and qPCR.

Supplementary 5 Table S1. List of specific primers of 15 differentially expressed mRNA designed utilizing primer 5.0.

## References

[1] Zhao W., Lei H., Zhu X., Li L., Qu S., Liang X. (2016). Investigation of long-term survival outcomes and failure patterns of patients with nasopharyngeal carcinoma receiving intensity-modulated radiotherapy: A retrospective analysis. *Oncotarget *.

[2] Ye W., Chang E. T., Liu Z. (2017). Development of a population-based cancer case-control study in southern china. *Oncotarget*.

[3] Sung W.-W., Chen P.-R., Liao M.-H., Lee J.-W. (2017). Enhanced aerobic glycolysis of nasopharyngeal carcinoma cells by Epstein-Barr virus latent membrane protein 1. *Experimental Cell Research*.

[4] Wang K., Ge Y., Ni C. (2017). Epstein-Barr virus-induced up-regulation of TCAB1 is involved in the DNA damage response in nasopharyngeal carcinoma. *Scientific Reports*.

[5] Lin J. C., Wang W. Y., Chen K. Y. (2004). Quantification of plasma Epstein-Barr virus DNA in patients with advanced nasopharyngeal carcinoma. *The New England Journal of Medicine*.

[6] Hou X., Zhang L., Zhao C. (2006). Prognostic impact of plasma Epstein-Barr virus DNA concentration on distant metastasis in nasopharyngeal carcinoma. *Ai Zheng*.

[7] Gurtsevitch V. E., Senyuta N. B., Ignatova A. V. (2017). Epstein-barr virus biomarkers for nasopharyngeal carcinoma in non-endemic regions. *Journal of General Virology*.

[8] Chen M., Yin L., Wu J. (2015). Impact of plasma epstein-barr virus-DNA and tumor volume on prognosis of locally advanced nasopharyngeal carcinoma. *BioMed Research International*.

[9] Lu L., Li J., Zhao C. (2016). Prognostic efficacy of combining tumor volume with Epstein-Barr virus DNA in patients treated with intensity-modulated radiotherapy for nasopharyngeal carcinoma. *Oral Oncology*.

[10] Costoya J. A. (2007). Functional analysis of the role of POK transcriptional repressors. *Briefings in Functional Genomics & Proteomics*.

[11] Maeda T., Hobbs R. M., Morghoub T. (2005). Role of the proto-oncogene Pokemon in cellular transformation and ARF repression. *Nature*.

[12] Zhao Z.-H., Wang S.-F., Yu L. (2008). Overexpression of Pokemon in non-small cell lung cancer and foreshowing tumor biological behavior as well as clinical results. *Lung Cancer*.

[13] Jiang L., Siu M. K., Wong O. G. (2010). Overexpression of proto-oncogene FBI-1 activates membrane type 1-matrix metalloproteinase in association with adverse outcome in ovarian cancers. *Molecular Cancer*.

[14] Qu H., Qu D., Chen F., Zhang Z., Liu B., Liu H. (2010). ZBTB7 overexpression contributes to malignancy in breast cancer. *Cancer Investigation*.

[15] Fang F., Yang L., Tao Y., Qin W. (2012). FBI-1 promotes cell proliferation and enhances resistance to chemotherapy of hepatocellular carcinoma in vitro and in vivo. *Cancer*.

[16] Zhang L., Wang Y., Zhang L. (2019). ZBTB7A, a miR-663a target gene, protects osteosarcoma from endoplasmic reticulum stress-induced apoptosis by suppressing LncRNA GAS5 expression. *Cancer Letters*.

[17] Wang G., Lunardi A., Zhang J. (2013). Zbtb7a suppresses prostate cancer through repression of a Sox9-dependent pathway for cellular senescence bypass and tumor invasion. *Nature Genetics*.

[18] Liu X.-S., Genet M. D., Haines J. E. (2015). Zbtb7a suppresses melanoma metastasis by transcriptionally repressing mcam. *Molecular Cancer Research*.

[19] Zhao Y., Yao Y., Li L. (2014). Pokemon enhances proliferation, cell cycle progression and anti-apoptosis activity of colorectal cancer independently of p14ARF–MDM2–p53 pathway. *Medical Oncology*.

[20] Liu X., Haines J. E., Mehanna E. K. (2014). ZBTB7A acts as a tumor suppressor through the transcriptional repression of glycolysis. *Genes & Development*.

[21] Shi D.-B., Wang Y.-W., Xing A.-Y. (2015). C/EBP*α*-induced miR-100 expression suppresses tumor metastasis and growth by targeting ZBTB7A in gastric cancer. *Cancer Letters*.

[22] Sun G., Peng B., Xie Q., Ruan J., Liang X. (2018). Upregulation of ZBTB7A exhibits a tumor suppressive role in gastric cancer cells. *Molecular Medicine Reports*.

[23] Jiao W., Liu F., Tang F.-Z. (2013). Expression of the pokemon proto-oncogene in nasopharyngeal carcinoma cell lines and tissues. *Asian Pacific Journal of Cancer Prevention*.

[24] Liu F., Tang F., Lan J. (2018). Stable knockdown of ZBTB7A promotes cell proliferation and progression in nasopharyngeal carcinoma. *Tumori*.

[25] Liu F., Lan J., Jiao W. (2017). Differences in Zbtb7a expression cause heterogeneous changes in human nasopharyngeal carcinoma CNE3 sublines. *Oncology Letters*.

[26] Wang K. C., Chang H. Y. (2011). Molecular mechanisms of long noncoding RNAs. *Molecular Cell*.

[27] Wen X., Tang X., Li Y. (2016). Microarray expression profiling of long non-coding RNAs involved in nasopharyngeal carcinoma metastasis. *International Journal of Molecular Sciences*.

[28] Nie Y., Liu X., Qu S., Song E., Zou H., Gong C. (2013). Long non-coding RNA *HOTAIR* is an independent prognostic marker for nasopharyngeal carcinoma progression and survival. *Cancer Science*.

[29] Bo H., Gong Z., Zhang W. (2015). Upregulated long non-coding RNA AFAP1-AS1 expression is associated with progression and poor prognosis of nasopharyngeal carcinoma. *Oncotarget *.

[30] Yang L., Tang Y., He Y. (2017). High Expression of LINC01420 indicates an unfavorable prognosis and modulates cell migration and invasion in nasopharyngeal carcinoma. *Journal of Cancer*.

[31] Zou Z. W., Ma C., Medoro L. (2016). LncRNA ANRIL is up-regulated in nasopharyngeal carcinoma and promotes the cancer progression via increasing proliferation, reprograming cell glucose metabolism and inducing sidepopulation stem-like cancer cells. *Oncotarget *.

[32] Shen M., Li M., Liu J. (2019). Long noncoding RNA HOTTIP promotes nasopharyngeal cancer cell proliferation, migration, and invasion by inhibiting miR-4301. *Medical Science Monitor*.

[33] Hao Y., Zhao H., Jin X. (2019). Long noncoding RNA DANCR promotes nasopharyngeal carcinoma cell proliferation and migration. *Molecular Medicine Reports*.

[34] Chak W.-P., Lung R. W.-M., Tong J. H.-M. (2017). Downregulation of long non-coding RNA MEG3 in nasopharyngeal carcinoma. *Molecular Carcinogenesis*.

[35] Guo J., Ma J., Zhao G. (2017). Long noncoding RNA LINC0086 functions as a tumor suppressor in nasopharyngeal carcinoma by targeting miR-214. *Oncology Research : Featuring Preclinical and Clinical Cancer Therapeutics*.

[36] Zhang L., Wang Y., Li X. (2017). ZBTB7A enhances osteosarcoma chemoresistance by transcriptionally repressing lncRNALINC00473-IL24 activity. *Neoplasia (United States)*.

[37] Zhao Z., Wang J., Wang S., Chang H., Zhang T., Qu J. (2017). LncRNA CCAT2 promotes tumorigenesis by over-expressed Pokemon in non-small cell lung cancer. *Biomedicine & Pharmacotherapy*.

[38] Gu S. Y., Tang W. P., Zeng Y. (1983). An epithelial cell line established from poorly differentiated nasopharyngeal carcinoma. *Ai Zheng*.

[39] Jiao W. (1995). Establishment of a human epithelial cell line of nasopharyngeal carcinoma – CNE3 and its biological characteristics. *Journal of Guangxi Medical University*.

[40] Liu F., Jiao W., Mo X.-L. (2013). Molecular pathological study of the human nasopharyngeal carcinoma CNE3 cell line. *Oncology Letters*.

[41] Chen W., Lee Y., Wang H. (1992). Suppression of human nasopharyngeal carcinoma cell growth in nude mice by the wild-type p53 gene. *Journal of Cancer Research and Clinical Oncology*.

[42] Teng Z.-P., Ooka T., Huang D. P., Zeng Y. (1996). Detection of Epstein-Barr Virus DNA in well and poorly differentiated nasopharyngeal carcinoma cell lines. *Virus Genes*.

[43] Faghihi M. A., Wahlestedt C. (2009). Regulatory roles of natural antisense transcripts. *Nature Reviews Molecular Cell Biology*.

[44] Ørom U. A., Derrien T., Beringer M. (2010). Long noncoding RNAs with enhancer-like function in human cells. *Cell*.

[45] Cabili M. N., Trapnell C., Goff L. (2011). Integrative annotation of human large intergenic noncoding RNAs reveals global properties and specific subclasses. *Genes & Development*.

[46] Wang Q., Li C., Tang P., Ji R., Chen S., Wen J. (2018). A minimal lncRNA-mRNA signature predicts sensitivity to neoadjuvant chemotherapy in triple-negative breast cancer. *Cellular Physiology and Biochemistry*.

[47] Su K., Zhao Q., Bian A., Wang C., Cai Y., Zhang Y. (2018). A novel positive feedback regulation between long noncoding RNA UICC and IL-6/STAT3 signaling promotes cervical cancer progression. *American Journal of Cancer Research*.

[48] Luan W., Zhou Z., Ni X. (2018). Long non-coding RNA H19 promotes glucose metabolism and cell growth in malignant melanoma via miR-106a-5p/E2F3 axis. *Journal of Cancer Research and Clinical Oncology*.

[49] Xiao Z., Han L., Lee H. (2017). Energy stress-induced lncRNA FILNC1 represses c-Myc-mediated energy metabolism and inhibits renal tumor development. *Nature Communications*.

[50] Liu X., Liang Y., Song R. (2018). Long non-coding RNA NEAT1-modulated abnormal lipolysis via ATGL drives hepatocellular carcinoma proliferation. *Molecular Cancer*.

[51] Christensen L. L., True K., Hamilton M. P. (2016). SNHG16 is regulated by the Wnt pathway in colorectal cancer and affects genes involved in lipid metabolism. *Molecular Oncology*.

[52] Ma D. D., Yuan L. L., Lin L. Q. (2017). LncRNA HOTAIR contributes to the tumorigenesis of nasopharyngeal carcinoma via up-regulating FASN. *European Review for Medical and Pharmacological Sciences*.

[53] Zhang H., Li Y., Wang H. (2018). Author Correction: Ephrin receptor A2 is an epithelial cell receptor for Epstein–Barr virus entry. *Nature Microbiology*.

[54] Zong D., Yin L., Zhong Q. (2016). ZNF488 enhances the invasion and tumorigenesis in nasopharyngeal carcinoma via the Wnt signaling pathway involving epithelial mesenchymal transition. *Cancer Research and Treatment*.

[55] Qi X.-K., Han H.-Q., Zhang H.-J. (2018). OVOL2 links stemness and metastasis via fine-tuning epithelial-mesenchymal transition in nasopharyngeal carcinoma. *Theranostics*.

[56] Mariya T., Hirohashi Y., Torigoe T. (2016). Matrix metalloproteinase-10 regulates stemness of ovarian cancer stem-like cells by activation of canonical Wnt signaling and can be a target of chemotherapy-resistant ovarian cancer. *Oncotarget *.

[57] Martin A., Salvador F., Moreno-Bueno G. (2015). Lysyl oxidase-like 2 represses Notch1 expression in the skin to promote squamous cell carcinoma progression. *EMBO Journal*.

[58] Yuan K., Lei Y., Chen H. (2016). HBV-induced ROS accumulation promotes hepatocarcinogenesis through Snail-mediated epigenetic silencing of SOCS3. *Cell Death & Differentiation*.

[59] Xu Z., Chen H., Liu D., Huo J. (2015). Fibulin-1 Is downregulated through promoter hypermethylation in colorectal cancer. *Medicine*.

[60] Sundararajan V., Gengenbacher N., Stemmler M. P., Kleemann J. A., Brabletz T., Brabletz S. (2015). The ZEB1/miR-200c feedback loop regulates invasion via actin interacting proteins MYLK and TKS5. *Oncotarget *.

[61] Flores-Téllez T. N. J., Lopez T. V., Vásquez Garzón V. R., Villa-Treviño S. (2015). Co-expression of Ezrin-CLIC5-Podocalyxin is associated with migration and invasiveness in hepatocellular carcinoma. *PLoS ONE*.

[62] Panneerselvam J., Jin J., Shanker M. (2015). IL-24 inhibits lung cancer cell migration and invasion by disrupting The SDF-1/CXCR4 signaling axis. *PLoS ONE*.

[63] Liu Y., Chen C., Xu Z. (2016). Deletions linked to TP53 loss drive cancer through p53-independent mechanisms. *Nature*.

[64] Liu R.-Z., Li S., Garcia E. (2016). Association between cytoplasmic CRABP2, altered retinoic acid signaling, and poor prognosis in glioblastoma. *Glia*.

[65] Yang Q., Wang R., Xiao W., Sun F., Yuan H., Pan Q. (2016). Cellular retinoic acid binding protein 2 is strikingly downregulated in human esophageal squamous cell carcinoma and functions as a tumor suppressor. *PLoS ONE*.

[66] Hsieh Y.-H., Hsieh S.-C., Lee C.-H. (2015). Targeting EMP3 suppresses proliferation and invasion of hepatocellular carcinoma cells through inactivation of PI3K/Akt pathway. *Oncotarget *.

[67] Xue Q., Zhou Y., Wan C. (2013). Epithelial membrane protein 3 is frequently shown as promoter methylation and functions as a tumor suppressor gene in non-small cell lung cancer. *Experimental and Molecular Pathology*.

[68] Yeo S. Y., Itahana Y., Guo A. K. (2016). Transglutaminase 2 contributes to a TP53-induced autophagy program to prevent oncogenic transformation. *eLife*.

[69] Leicht D. T., Kausar T., Wang Z. (2014). TGM2: A cell surface marker in esophageal adenocarcinomas. *Journal of Thoracic Oncology*.

[70] Lehmann W., Mossmann D., Kleemann J. (2016). ZEB1 turns into a transcriptional activator by interacting with YAP1 in aggressive cancer types. *Nature Communications*.

[71] Yuan M., Tomlinson V., Lara R. (2008). Yes-associated protein (YAP) functions as a tumor suppressor in breast. *Cell Death & Differentiation*.

[72] Cheung C. T., Singh R., Kalra R. S., Kaul S. C., Wadhwa R. (2014). Collaborator of ARF (CARF) regulates proliferative fate of human cells by dose-dependent regulation of DNA damage signaling. *The Journal of Biological Chemistry*.

[73] Huang Y.-Y., Dai L., Gaines D. (2013). BCCIP suppresses tumor initiation but is required for tumor progression. *Cancer Research*.

[74] Kao Y.-C., Lee S.-W., Lin L.-C. (2013). Fatty acid synthase overexpression confers an independent prognosticator and associates with radiation resistance in nasopharyngeal carcinoma. *Tumor Biology*.

[75] Bochenek G., Häsler R., Mokhtari N.-E. E. (2013). The large non-coding RNA ANRIL, which is associated with atherosclerosis, periodontitis and several forms of cancer, regulates ADIPOR1, VAMP3 and C11ORF10. *Human Molecular Genetics*.

[76] Wu J., Tang J., Li J., Li X. (2018). Upregulation of SOX2-activated lncRNA ANRIL promotes nasopharyngeal carcinoma cell growth. *Scientific Reports*.

[77] Hu X., Jiang H., Jiang X. (2017). Downregulation of lncRNA ANRIL inhibits proliferation, induces apoptosis, and enhances radiosensitivity in nasopharyngeal carcinoma cells through regulating miR-125a. *Cancer Biology & Therapy*.

[78] Wang Y., Cheng N., Luo J. (2017). Downregulation of lncRNA ANRIL represses tumorigenicity and enhances cisplatin-induced cytotoxicity via regulating microRNA let-7a in nasopharyngeal carcinoma. *Journal of Biochemical and Molecular Toxicology*.

[79] Guo D., Bell E. H., Mischel P., Chakravarti A. (2014). Targeting SREBP-1-driven lipid metabolism to treat cancer. *Current Pharmaceutical Design*.

[80] Lo A. K.-F., Lung R. W.-M., Dawson C. W. (2018). Activation of sterol regulatory element-binding protein 1 (SREBP1)-mediated lipogenesis by the Epstein–Barr virus-encoded latent membrane protein 1 (LMP1) promotes cell proliferation and progression of nasopharyngeal carcinoma. *The Journal of Pathology*.

[81] Wu M. X., Ustyugova I. V., Han L., Akilov O. E. (2013). Immediate early response gene X-1, a potential prognostic biomarker in cancers. *Expert Opinion on Therapeutic Targets*.

[82] Ye J., Zhang Y., Cai Z. (2018). Increased expression of immediate early response gene 3 protein promotes aggressive progression and predicts poor prognosis in human bladder cancer. *BMC Urology*.

[83] Han L., Geng L., Liu X., Shi H., He W., Wu M. X. (2011). Clinical significance of IEX-1 expression in ovarian carcinoma. *Ultrastructural Pathology*.

[84] Wang H. Y., Li F., Liu N. (2019). Prognostic implications of a molecular classifier derived from whole-exome sequencing in nasopharyngeal carcinoma. *Cancer Medicine*.

[85] Chen R.-H., Du Y., Han P. (2016). ISG15 predicts poor prognosis and promotes cancer stem cell phenotype in nasopharyngeal carcinoma. *Oncotarget *.

