# Supplementary figures and images for: Long Noncoding RNAs and Messenger RNAs Expression Profiles Potentially Regulated by ZBTB7A in Nasopharyngeal Carcinoma
Source: Biomed Res Int. 2019 Jun 11;2019:7246491. doi: 10.1155/2019/7246491 (PMC6594332; doi:10.1155/2019/7246491)

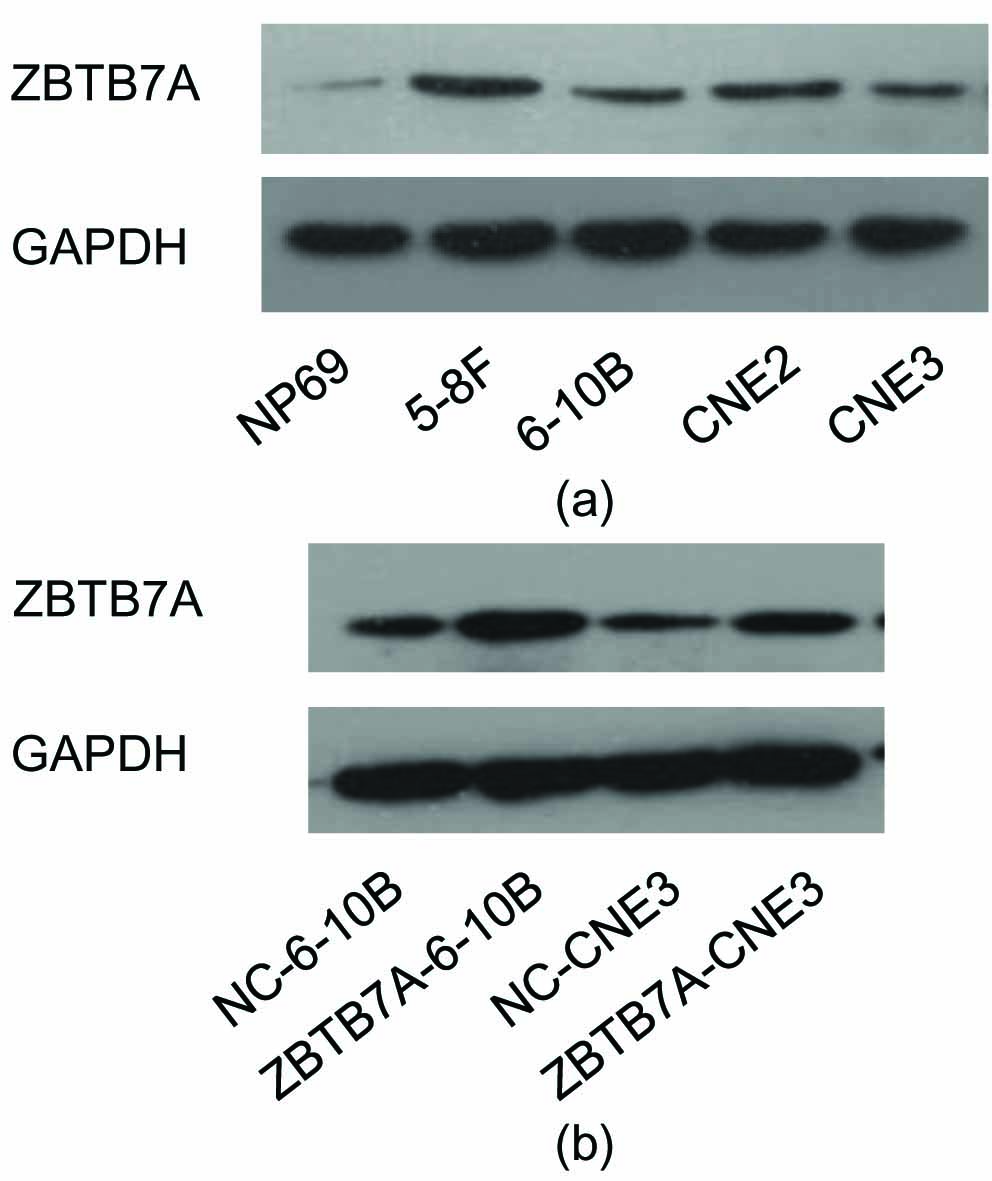

Supplement: Supplementary 1 — Figure S1. The protein levels of ZBTB7A in NPC cell lines. [file 7246491.f1.jpg]

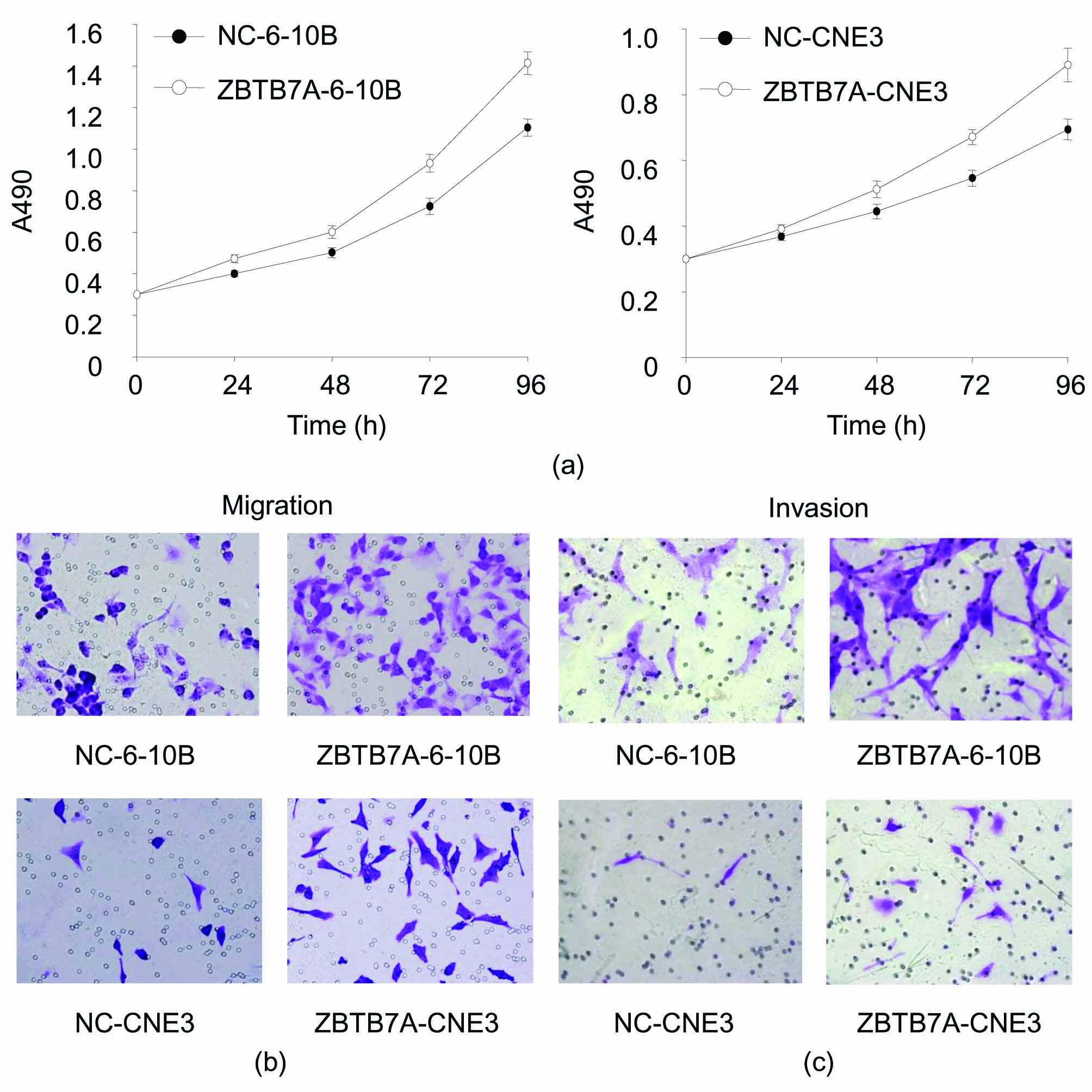

Supplement: Supplementary 2 — Figure S2. The vitality, migration, and invasion of 6-10B and CNE3 stably transfected cells with ZBTB7A overexpression. [file 7246491.f2.jpg]

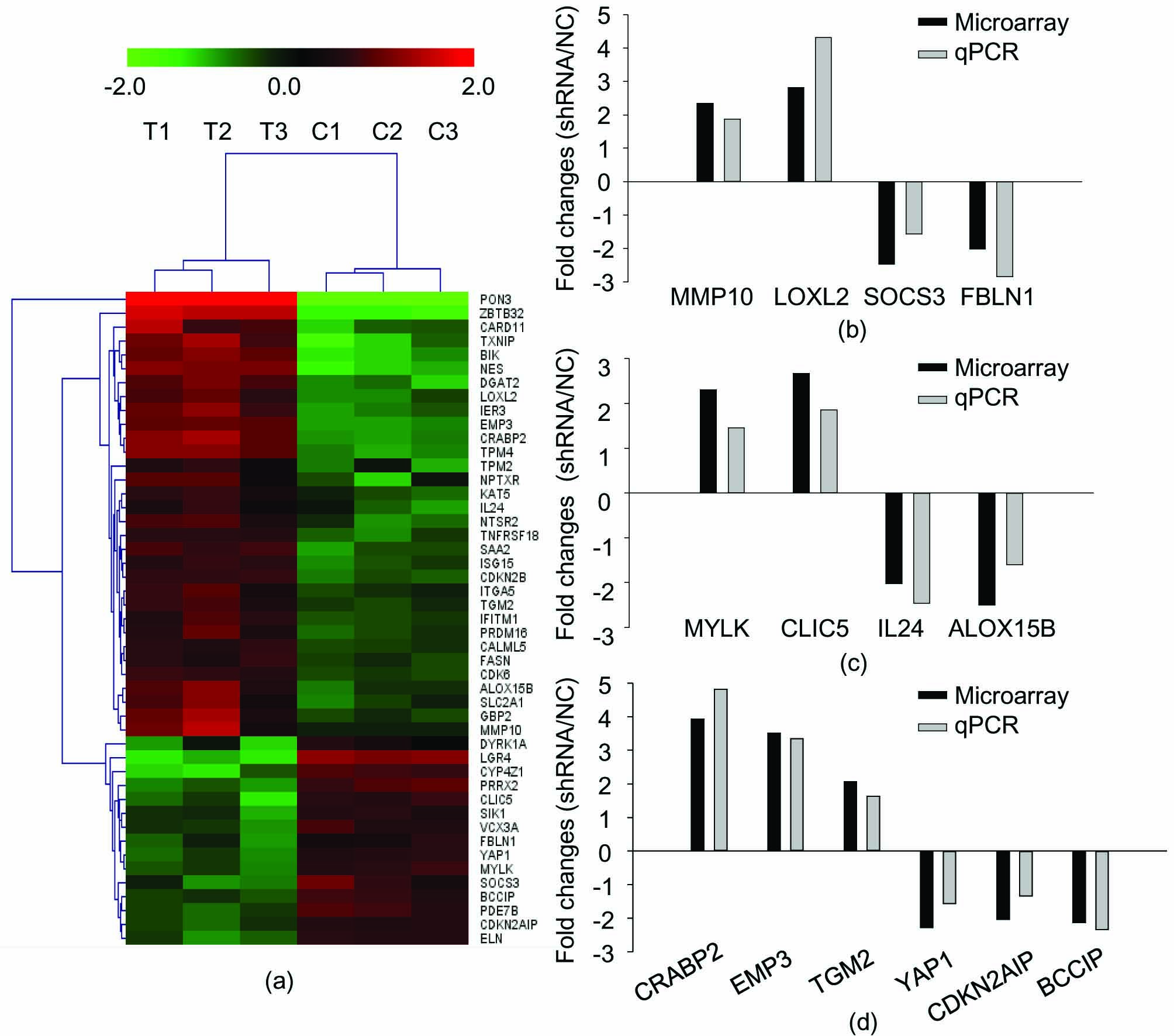

Supplement: Supplementary 3 — Figure S3. Screening and validation of 14 differentially expressed mRNAs by microarray and qPCR. [file 7246491.f3.jpg]

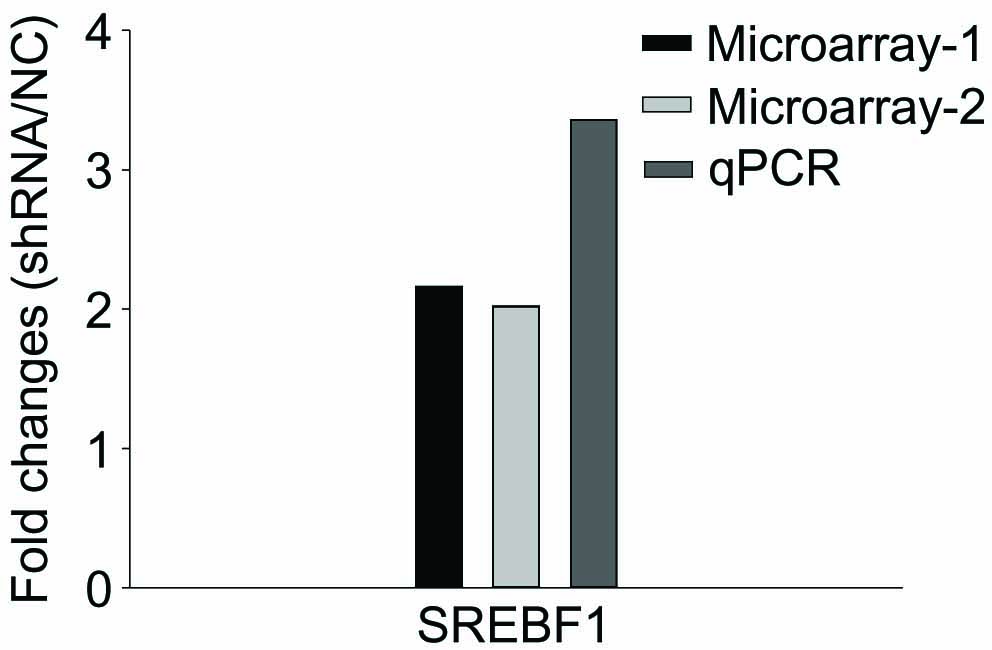

Supplement: Supplementary 4 — Figure S4. Screening and validation of differentially expressed SREBF1 by microarray and qPCR. [file 7246491.f4.jpg]
