# Supplementary material for: Long Noncoding RNAs and Messenger RNAs Expression Profiles Potentially Regulated by ZBTB7A in Nasopharyngeal Carcinoma
Source: Biomed Res Int. 2019 Jun 11;2019:7246491. doi: 10.1155/2019/7246491 (PMC6594332; doi:10.1155/2019/7246491)
Supplement: Supplementary 5 — Table S1. List of specific primers of 15 differentially expressed mRNA designed utilizing primer 5.0. [file 7246491.f5.pdf]

Supplementary 5. Table S1

TABLE S1: The list of specific primers of 15 differentially expressed mRNAs designed utilizing primer 5.0

Gene Symbol	Sequence name	Prime Sequence
GAPDH*	NM_002046.5	F:5’GGGAAACTGTGGCGTGAT3’
(Human)		R:5’GAGTGGGTGTCGCTGTTGA3’
MMP10	NM_002425.2	F:5’CGTTGGTCACTTCAGCTCCT3’
		R:5’TCAATGGCAGAATCAACAGC3’
LOXL2	NM_002318.2	F:5’CATCCACCTCAACGAGATCC3’
		R:5’TCTCACACCAGCATCCTCCT3’
SOCS3	NM_003955.4	F:5’CTGGTGGTGAACGCAGTG3’
		R:5’AGCTGTCGCGGATCAGAA3’
FBLN1	NM_001996.3	F:5’CATCAACGAGACCTGCTTCA3’
		R:5’GATTCTCATGGCAAGGCAAG3’
MYLK	NM_053027.3	F:5’CAGTGCCAGGTGTCTTCTGA3’
		R:5’GAGCAGAGTGAGCCTTCCTG3’
CLIC5	NM_016929.4	F:5’CCATGTGGTCAAGATTGTGG3’
		R:5’TGCACAGGTGTTGGTGAACT3’
IL24	NM_001185156.1	F:5’CTCGGATGCTGAGAGCTGTT3’
		R:5’GGCCAGAGTAGAGAATGACTTCAG3’
ALOX15B	NM_001039131.1	F:5’CCGAGGAGTTGAAGACATCC3’
		R:5’GGCGGAGCAGGTGAATATC3’
CRABP2	NM_001878.3	F:5’CAACTTCTCTGGCAACTGGA3’
		R:5’GATCTCCACTGCTGGCTTG3’
EMP3	ENST00000270221	F:5’GCCATGTCACTCCTCTTGCT3’
		R:5’ACGTGCAGTCGTACCAGAGA3’
TGM2	NM_004613.3	F:5’ACCGCTGAGGAGTACGTCTG3’
		R:5’CAGGTTGAGGTTGAGCAGGT3’
YAP1	NM_001195044.1	F:5’AGACAACAACATGGCAGGAC3’

		R:5’CCATCCATCAGGAAGAGGAC3’
BCCIP	NM_016567.3	F:5’CCAGTTCCGATACAGCACAA3’
		R:5’GACAGAGCAATTCCAACAGTCA3’
CDKN2AIP	NM_017632.3	F:5’AGCTCTGGCATCTCCAGTCA3’
		R:5’CAGATCCTGCCGTTGTTACC3’
SREBF1	NM_004176 and	F:5’ GCTGTTGGTGCTCGTCTCCTTG3’
	NM_001005291	R:5’ GCTTGCGATGCCTCCAGAAGTAC3’

* GAPDH was used to as an internal reference control.
